# Crystallization and X-ray analysis of 23 nm virus-like particles from *Norovirus* Chiba strain

**DOI:** 10.1107/S2053230X17013759

**Published:** 2017-10-02

**Authors:** Kazuya Hasegawa, Yuichi Someya, Hideki Shigematsu, Tomomi Kimura-Someya, Nipawan Nuemket, Takashi Kumasaka

**Affiliations:** a Protein Crystal Analysis Division, Japan Synchrotron Radiation Research Institute, 1-1-1 Kouto, Sayo, Hyogo 679-5198, Japan; b Department of Virology II, National Institute of Infectious Diseases, 4-7-1 Gakuen, Musashi-Murayama, Tokyo 208-0011, Japan; c Division of Structural and Synthetic Biology, RIKEN Center for Life Science Technologies, 1-7-22 Suehiro-cho, Tsurumi-ku, Yokohama, Kanagawa 230-0045, Japan

**Keywords:** *Norovirus*, GI.4 Chiba strain, virus-like particles, crystallization, 23 nm VLPs

## Abstract

*Norovirus* is a major causative pathogen of nonbacterial acute gastroenteritis. Despite the sequence similarity among various strains, noroviruses of different genotypes show different antigenicities and different binding profiles to histo-blood group antigens (HBGAs). To reveal the relationships between the structure of the capsid and the diversity in antigenicity and the HBGA-binding profile, virus-like particles (VLPs) of the Chiba strain that belongs to genogroup I, genotype 4 were crystallized for X-ray structural analysis. Diffraction data were collected and processed at 3.2 Å resolution. The crystal belonged to space group *I*222, with unit-cell parameters *a* = 290.0, *b* = 310.4 *c* = 350.4 Å. The possible packing model indicated that the diameter of the particle was 280 Å, which was much smaller than the 38 nm VLPs of *Norovirus* Norwalk strain (NV) with *T* = 3 icosahedral symmetry and composed of 180 VP1 proteins. The structure was solved by molecular replacement using the structure of the VP1 pentamer of NV 38 nm VLPs as a search model, revealing that the VLPs were smaller particles: 23 nm VLPs with *T* = 1 icosahedral symmetry, the structure of which has not yet been analyzed at high resolution. The structure of 23 nm VLPs will enable the two different VLPs of *Norovirus* to be compared, which will provide important information for understanding the structural basis of capsid formation.

## Abstract 2

Virus-like particles (VLPs) from *Norovirus* Chiba strain were crystallized and data were collected to 3.2 Å resolution. X-ray analysis revealed that although 38 nm VLPs with *T* = 3 symmetry had been prepared for crystallization, the crystal contained 23 nm VLPs with *T* = 1 icosahedral symmetry.

## 1. Introduction   

*Norovirus* is a positive-sense, single-stranded RNA virus belonging to the *Caliciviridae* family. It is well known as a causative pathogen of nonbacterial acute gastroenteritis in humans. Based on sequence diversity, human noroviruses are divided into two major genogroups, GI and GII, in which at least nine and 22 genotypes have been identified, respectively (Kroneman *et al.*, 2013 ▸). Its 7.7 kb genome has three ORFs: ORF1 encoding a polyprotein precursor of the nonstructural proteins, ORF2 encoding the major structural protein VP1 and ORF3 encoding the minor structural protein VP2 that is thought to interact with viral RNA. A virus capsid is made by 180 copies of VP1 self-assembling to form a *T* = 3 icosahedral shell. Expressed in insect cells, VP1 self-assembles into empty virus-like particles (VLPs) of 38 nm in diameter. Because these VLPs are structurally and antigenically similar to the intact viruses, they have been used as immunogens in vaccine development. There are also smaller VLPs of 23 nm in diameter that consist of 60 copies of VP1 and have *T* = 1 icosahedral symmetry (White *et al.*, 1997 ▸).

The crystal structure of 38 nm VLPs from the GI *Norwalk virus* (NV; GI.1) was solved by Prasad *et al.* (1999 ▸), revealing that the VP1 protein consists of two domains: an S domain that forms contiguous spherical shells with an inner radius of 100 Å and an outer radius of 145 Å, and a P domain that forms a protrusion from the spherical shells by dimerization with the P domain of a neighbouring subunit. The surface loop at the tip of a protruding P domain contributes to the recognition of histo-blood group antigens (HBGAs). The crystal structure of the P domain in complex with HBGAs revealed that the binding sites of GI and GII strains were different (Choi *et al.*, 2008 ▸). Moreover, even within a single genogroup the longer surface loops of the Funabashi 258 strain (GI.2) compared with those of the Norwalk strain (GI.1) result in a different mode of carbohydrate binding (Kubota *et al.*, 2012 ▸). These differences may make it difficult to develop a vaccine targeting a wide range of strains.

*Norovirus* Chiba strain (ChV; GI.4) was identified as the cause of an oyster-associated outbreak of gastroenteritis in Chiba prefecture, Japan in 1987 (Kobayashi *et al.*, 2000 ▸). The entire RNA genome of ChV has been cloned and sequenced (Someya *et al.*, 2000 ▸), and the structure of the 3C protease of ChV has been determined (Nakamura *et al.*, 2005 ▸). The sequence similarity of ChV VP1 to NV VP1 is 85% in the S domain and 64% in the P domain. Although the overall structure of ChV VLPs is thought to be similar to that of NV VLPs, the different HBGA-binding profiles of ChV and NV VLPs suggest that there is a structural difference between these VLPs (Shirato *et al.*, 2008 ▸). Understanding this structural diversity is important for the development of a vaccine targeting various strains, which encouraged us to analyze the structure of ChV VLPs using X-ray crystallography.

Crystals of ChV VLPs were obtained using 38 nm VLPs of triple-mutant ChV VP1 (Someya *et al.*, 2011 ▸). Diffraction data were collected and processed to 3.2 Å resolution. Although our crystals of ChV VLPs were obtained using 38 nm VLPs, molecular replacement revealed that our crystals contained 23 nm VLPs.

## 2. Materials and methods   

### 2.1. Macromolecule production   

The VLPs used in this study were triple-mutant VLPs in which the original Leu43-Ala44-Thr45 sequence of the ChV VP1 had been converted to Ala-Pro-Val to prevent cleavage during expression and purification (Someya *et al.*, 2011 ▸). These residues are located near the end of the N-terminal arm preceding the S domain and are involved in intermolecular interaction (Prasad *et al.*, 1999 ▸); digestion of this N-terminal arm is known to lead to the formation of 23 nm VLPs (White *et al.*, 1997 ▸; Someya *et al.*, 2011 ▸).

**Figure 1:**
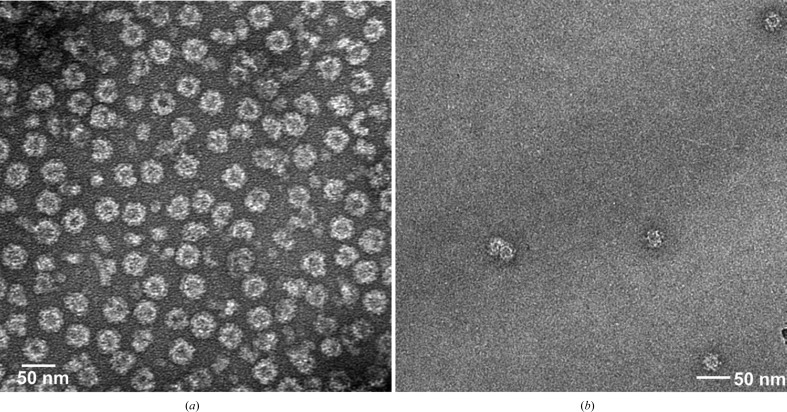
Negative-stained electron micrographs of purified ChV VLPs. (*a*) Purified VLPs. (*b*) After dissolving crystals in buffer solution.

VLPs were prepared as described in Someya *et al.* (2011 ▸). The transfer vector pORBCVORF2,3-LAT2APV encoding the triple-mutant VP1 protein and VP2 was introduced into Sf9 insect cells (Invitrogen) with baculovirus DNA to obtain recombinant baculoviruses. The recombinant baculoviruses were then used to infect HighFive (Tn5) insect cells (Invitrogen) to express VLPs. After cultivation for one week, VLPs were collected from the medium by ultracentrifugation and were purified by CsCl density-gradient centrifugation. Because negative-staining electron microscopy (EM) showed 38 nm VLPs together with partially destroyed VLPs and particle-like structures whose diameter was much smaller than that of 23 nm VLPs, the collected VLPs were further purified by sucrose density-gradient centrifugation, which removed most of these contaminants (Fig. 1 ▸ *a*). Macromolecule-production information is shown in Table 1 ▸.

### 2.2. Crystallization   

**Figure 2:**
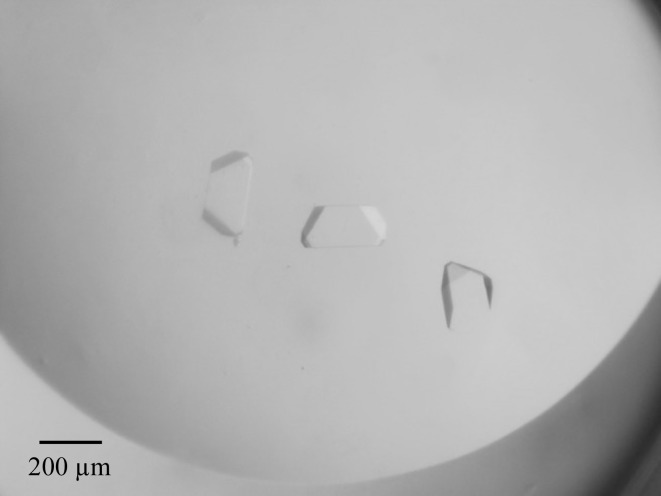
Crystals of ChV VLPs crystallized in 20 m*M* (NH_4_)_2_HPO_4_, 1% PEG 10 000.

VLPs were crystallized at 293 K in 96-well plates using the sitting-drop vapour-diffusion method. Prior to crystallization, the buffer solution of purified VLPs was replaced with 20 m*M* MES pH 6.5 and they were concentrated to 4 mg ml^−1^. Initial screening was performed using PEG/Ion Screen (Hampton Research), which was diluted fivefold and tenfold because the crystallization-condition information in VIPERdb (Carrillo-Tripp *et al.*, 2009 ▸) shows that many viruses tend to crystallize in a combination of low concentrations of PEG and salt. Crystals of ChV VLPs were obtained under several conditions, and the largest crystals were obtained in the presence of dibasic phosphate ions. However, these crystals did not have sharp edges and appeared to be clusters of several crystals. Using a microseeding technique and optimizing the concentrations of PEG and (NH_4_)_2_HPO_4_ and the molecular weight of PEG resulted in the growth of large single crystals (Fig. 2 ▸). Higher concentrations of (NH_4_)_2_HPO_4_ or PEG tended to cause the formation of aggregates. The final crystallization condition is shown in Table 2 ▸. The speed of crystal growth was quite slow; it took one to three months to obtain crystals of 100 µm in size.

### 2.3. Data collection and processing   

**Figure 3:**
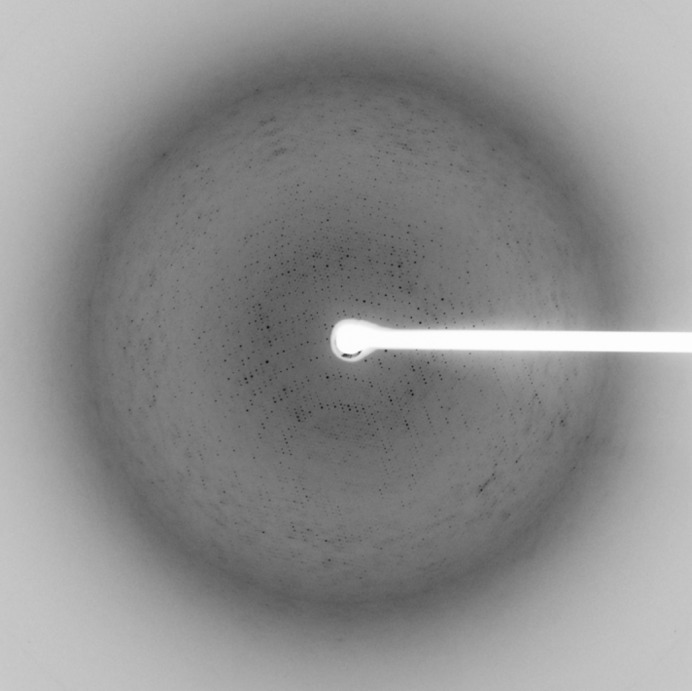
Diffraction image of a ChV VLP crystal.

Prior to data collection, the mother liquor of the crystals was replaced with 25%(*v*/*v*) glycerol, 20 m*M* (NH_4_)_2_HPO_4_, 2%(*w*/*v*) PEG 10 000 and the crystals were cooled in liquid nitrogen. All data were collected on BL41XU at SPring-8 (Hasegawa *et al.*, 2013 ▸) with a wavelength of 1.0 Å under a 100 K cold stream using an MX225HE CCD detector (Rayonix) (Fig. 3 ▸). The beam size was adjusted by pinhole collimation to either 30 or 50 µm depending on the crystal size, and helical scanning was utilized to mitigate radiation damage. Since there were saturated pixels up to 9 Å resolution in the measurement conditions for high-resolution data, low-resolution data were collected separately. Radiation damage made it difficult to obtain complete data from one crystal, so a total of 26 data sets were collected using 11 crystals (Supplementary Table S1). During the collection of high-resolution data, thin aluminium attenuators (≤300 µm) and small rotation steps (0.1–0.25° per frame) were used in order to increase the signal-to-noise ratio. Thicker aluminium attenuators (300–700 µm) and larger rotation steps (≥0.25° per frame) were used for the collection of low-resolution data. When the crystals were large enough, both high- and low-resolution data covering the same rotation range were collected from the same crystal.

Diffraction data were integrated with *XDS* (Kabsch, 2010 ▸). The saturated reflections were excluded from the output intensity files by using the OVERLOAD parameter in *XDS*. Cluster analysis was then performed with *BLEND* (Foadi *et al.*, 2013 ▸). The high-resolution cutoff of each data set was estimated by setting the *BLEND* keyword ISIGI to 1.5. Finally, 19 data sets from eight crystals with a linear cell variation of 0.63 were scaled and merged by *AIMLESS* (Evans & Murshudov, 2013 ▸). The data showed anisotropy: CC_1/2_ in the a* and c* directions decreased to 0.4 at 3.20 and 3.15 Å resolution, respectively, whereas in the b* direction it decreased to 0.4 at 3.65 Å resolution. Data statistics are shown in Table 3 ▸, where the resolution was cut at 3.2 Å using an overall CC_1/2_ of 0.4 as a threshold.

## 3. Results and discussion   

**Figure 4:**
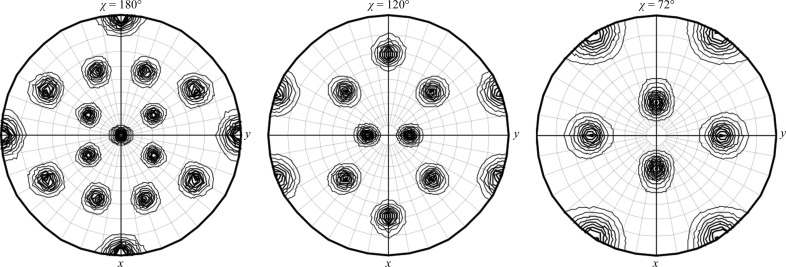
Self-rotation function for ChV VLPs in χ = 180, 120 and 72° sections calculated by *MOLREP* (Vagin & Teplyakov, 2010 ▸) with an integration radius of 40 Å in the resolution range 49.4–8 Å.

To obtain the capsid structure of ChV, we crystallized ChV VLPs. Diffraction data were collected and processed at 3.2 Å resolution using multiple crystals grown in 10–30 m*M* (NH_4_)_2_HPO_4_, 1–3% PEG 10 000. Data processing showed that the crystals belonged to space group *I*222 or *I*2_1_2_1_2_1_, with unit-cell parameters *a* = 290.0, *b* = 310.4, *c* = 350.4 Å. The self-rotation function in Fig. 4 ▸ shows peaks in the χ = 180, 120 and 72° sections, indicating the presence of icosahedral symmetry. The structure analysis was performed assuming space group *I*222 because to the best of our knowledge there are no virus structures in space group *I*2_1_2_1_2_1_. In the plausible VLP packing in this space group, there were eight particles at the corners of an orthorhombic cell and one particle at the centre. However, the largest allowable particle diameter in this packing model was 280 Å, which was much smaller than the size of 38 nm VLPs. To solve the structure, molecular replace­ment (MR) was performed with *Phaser* (McCoy *et al.*, 2007 ▸) using the VP1 pentamer of NV as a search model, which gave three solutions. The success of MR confirmed that the space group was *I*222. The model obtained by MR showed that the three pentamers were arranged around a local threefold axis. These three pentamers formed *T* = 1 icosahedral capsid shells together with nine pentamers related by crystallographic symmetry. This suggested that our crystal contained 23 nm VLPs of ChV, which was confirmed by EM observation of crystals dissolved in buffer solution (Fig. 1 ▸ *b*).

As mentioned in §[Sec sec2.1]2.1, the triple mutant of ChV VP1 was used to prevent N-terminal digestion and the formation of 23 nm VLPs, but the crystal unexpectedly contained 23 nm VLPs. There are two possible reasons why 38 nm VLP crystals could not be obtained. The first is freezing and thawing of the VLP solution. The purified VLPs were stored in a freezer until a sufficient amount was accumulated because the yield of the VLPs in one preparation was not sufficient for crystallization screening. The thawing of frozen VLPs for crystallization might physically disturb their structural integrity. The second possible reason is the pH of our crystallization condition. Ausar *et al.* (2006 ▸) reported that 38 nm VLPs were stable at pH 3–7 but became unstable at pH 8, which led to breakage and the formation of smaller particles. The pH of the reservoir solution was 7.9, which might cause instability of the VLPs. Although adding VLPs dissolved in 20 m*M* MES pH 6.5 decreased the pH to 7.1, the higher local pH immediately after they were added might have reduced their stability. Either the freezing and thawing or the elevated local pH, or both, might damage 38 nm VLPs.

**Figure 5:**
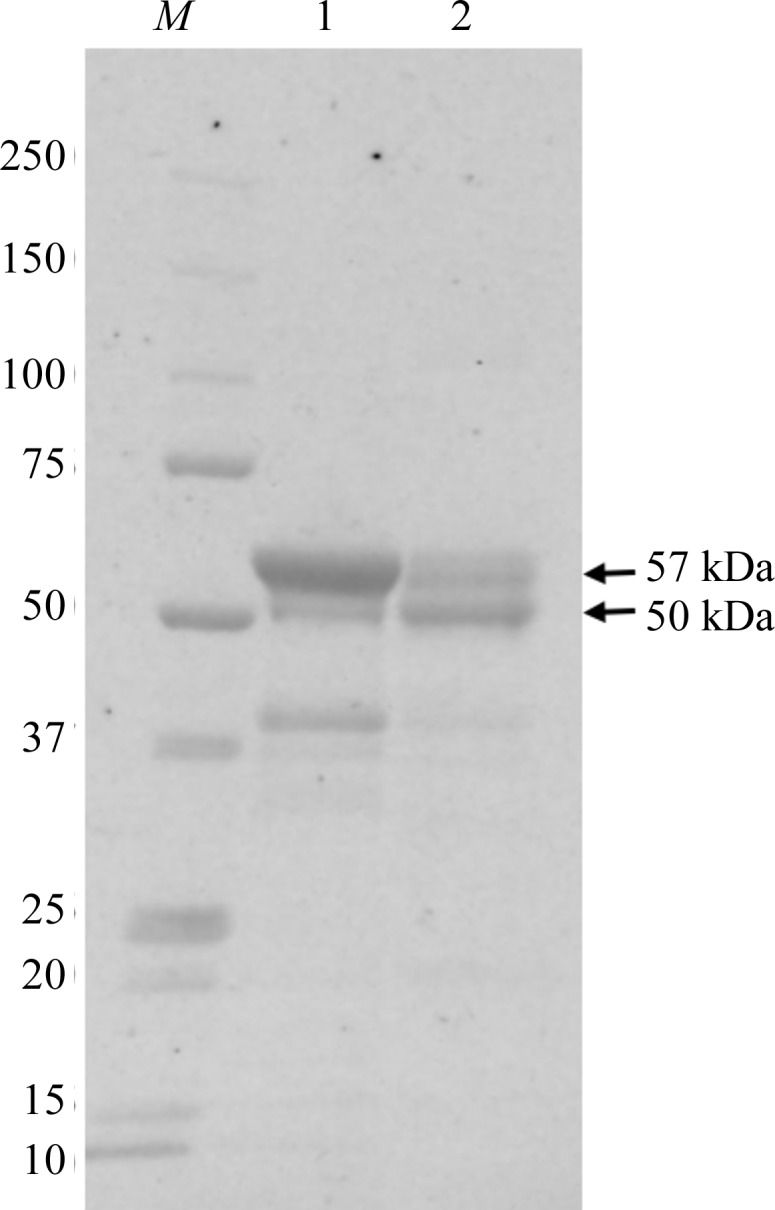
SDS–PAGE analysis of dissolved crystal (lane 2) and the VLP solution used for crystallization (lane 1). Four crystals stored in liquid nitrogen for diffraction data collection were dissolved in 10 µl 20 m*M* MES pH 6.5 prior to SDS–PAGE performed using 5–20% gradient gels stained with Oriole fluorescent gel stain (Bio-Rad). The 57 kDa band is the full-length VP1 and the 50 kDa band is VP1 with the N-terminal arm digested. The source of the band near 37 kDa is not known; it is larger than both the S-­domain and P-domain fragments. The 50 and 37 kDa bands in the crystallization solution were partly caused by VLP breakage owing to freezing and thawing. Lane *M* contains molecular-mass markers (labelled in kDa).

To find out whether or not the N-terminal residues of VP1 were digested in our crystals, we used sodium dodecyl sulfate–polyacrylamide gel electrophoresis (SDS–PAGE) to analyse the crystals after they had been dissolved in the buffer. The results (Fig. 5 ▸) showed that the VP1 in the crystal contained a 50 kDa fragment together with the 57 kDa intact protein, meaning that N-terminal arm of some of the VP1 protein in the crystal had been digested. The results also showed that the VLP solution before crystallization also contained VP1 with the N-terminal arm digested. This is partly caused by VLP breakage owing to freezing and thawing. However, the amount of digested VP1 in our crystal was much larger than that in the 38 nm VLP solution used for crystallization, which implies that the formation of 23 nm VLPs was owing to digested VP1. When the N-terminal arm of VP1 was digested remains unclear. One possibility is that a small amount of VP1 had already been digested during the preparation of the VLPs, which led to contamination of the purified 38 nm VLP solution with a trace amount of 23 nm VLPs. Another possibility is that the breakage of 38 nm VLPs made the VP1 susceptible to N-terminal digestion and 23 nm VLPs were formed during the long crystallization period.

The crystal structure of 38 nm NV VLPs showed that the N-terminal residues tended to form two distinct dimer conformations in VLPs: ‘bent’ *A*/*B* dimers and ‘flat’ *C*/*C* dimers (Prasad *et al.*, 1999 ▸). Although analysis of the structure is still in progress, the structure of 23 nm VLPs did not show electron density for the N-terminal 50 residues; this was partly caused by digestion of the N-terminal residues and partly caused by fluctuation of the conformation. Moreover, the dimer conformation in 23 nm VLPs was not that of the *A*/*B* or *C*/*C* dimers in 38 nm VLPs. The formation of *T* = 1 VLPs by the digestion of N-terminal residues has also been reported for *Hepatitis E virus* (HEV; Yamashita *et al.*, 2009 ▸; Guu *et al.*, 2009 ▸; Xing *et al.*, 2010 ▸) and *Grouper nervous necrosis virus* (GNNV; Chen *et al.*, 2015 ▸). In the case of HEV, the N-terminal 111 residues form an electropositive N domain, and interaction of the N domain with RNA makes the *C*/*C* dimer adopt a flat conformation (Xing *et al.*, 2010 ▸). In the case of GNNV, the flexible N-terminal arginine-rich motif (N-ARM) plays an important role in forming *T* = 3 VLPs (Chen *et al.*, 2015 ▸). On the other hand, the N-terminal arm of NV VP1 possesses only one basic residue and interaction between the N-terminal arm and RNA was not observed in the structure of NV 38 nm VLPs (Prasad *et al.*, 1999 ▸). Therefore, the mechanism by which NV VP1 proteins form 23 nm VLPs might differ from those by which HEV and HEV capsid proteins make smaller *T* = 1 VLPs.

It should be noted that particles of 23 nm in diameter have been isolated in stool samples from norovirus-infected patients (Taniguchi *et al.*, 1981 ▸) and were also produced in the norovirus replication system using stem cell-derived human enteroids (Ettayebi *et al.*, 2016 ▸), although the physiological role of the smaller particles is still unknown. We will be able to reveal the first high-resolution structure of 23 nm VLPs from a calicivirus. Comparison of their structure with that of 38 nm VLPs would provide important information for understanding the structural basis of capsid formation.

## Supplementary Material

Data-collection condition of 26 data sets.. DOI: 10.1107/S2053230X17013759/nw5058sup1.pdf

## Figures and Tables

**Table 1:** Macromolecule-production information

Source organism	*Norovirus* Chiba strain (GI.4)
DNA source	cDNA
Forward primer	TGACCTCGGATTGTGGACAG
Reverse primer	TTTTTTTTTTTAACATCAACCAAATCAAAATTAAAA
Cloning vector	pUC118
Expression vector	pORB
Expression host	Baculovirus and HighFive insect cells
Complete amino-acid sequence of the construct produced	MMMASKDATPSADGATGAGQLVPEVNTADPIPIDPVAGSSTAAPVAGQVNLIDPWIINNFVQAPQGEFTISPNNTPGDVLFDLQLGPHLNPFLSHLSQMYNGWVGNMRVRVVLAGNAFTAGKVIICCVPPGFQSRTLSIAQATLFPHVIADVRTLDPVEVPLEDVRNVLYHNNDTQPTMRLLCMLYTPLRTGGASGGTDSFVVAGRVLTCPGPDFNFLFLVPPTVEQKTRPFTVPNIPLKYLSNSRIPNPIEGMSLSPDQTQNVQFQNGRCTIDGQPLGTTPVSVSQLCKFRGRITSGQRVLNLTELDGSPFMAFAAPAPAGFPDLGSCDWHIEMSKIPNSSTQNNPIVTNSVKPNSQQFVPHLSSITLDENVSSGGDYIGTIQWTSPPSDSGGANTNFWKIPDYGSSLAEASQLAPAVYPPGFNEVIVYFMASIPGPNQSGSPNLVPCLLPQEYITHFISEQAPIQGEAALLHYVDPDTNRNLGEFKLYPGGYLTCVPNSSSTGPQQLPLDGVFVFASWVSRFYQLKPVGTAGPARGRLGVRR

**Table 2:** Crystallization

Method	Sitting-drop vapour diffusion
Plate type	96-well type, MRC
Temperature (K)	293
Protein concentration (mg ml^−1^)	4
Buffer composition of protein solution	20 m*M* MES pH 6.5
Composition of reservoir solution	10, 20 or 30 m*M* (NH_4_)_2_HPO_4_, 1, 2 or 3% PEG 10 000 pH 7.9
Volume and ratio of drop	2 µl, 1:1
Volume of reservoir (µl)	100

**Table 3:** Data collection and processing Values in parentheses are for the outer shell.

Diffraction source	BL41XU, SPring-8
Wavelength (Å)	1.0000
Temperature (K)	100
Detector	Rayonix MX225HE
Space group	*I*222
*a*, *b*, *c* (Å)	290.0, 310.4, 350.4
Mosaicity (°)	0.14
Resolution range (Å)	49.41–3.20 (3.25–3.20)
Total No. of reflections	6785578 (275713)
No. of unique reflections	257670 (12599)
Completeness (%)	99.9 (99.5)
Multiplicity	26.3 (21.9)
〈*I*/σ(*I*)〉	10.8 (2.9)
*R*_r.i.m._	0.455 (2.296)
CC_1/2_	0.99 (0.423†)
Overall *B* factor from Wilson plot (Å^2^)	71.5

† CC_1/2_ value obtained by curve fitting.
