# Supplementary material for: Crystallization and X-ray analysis of 23 nm virus-like particles from Norovirus Chiba strain
Source: Acta Crystallogr F Struct Biol Commun. 2017 Oct 2;73(Pt 10):568–73. doi: 10.1107/S2053230X17013759 (PMC5633924; doi:10.1107/S2053230X17013759)
Supplement: Supplementary file 1 [file f-73-00568-sup1.pdf]

STRUCTURAL BIOLOGY COMMUNICATIONS

Volume 73 (2017)

Supporting information for article:

Crystallization and X-ray analysis of 23 nm virus-like particles from *Norovirus* Chiba strain

Kazuya Hasegawa, Yuichi Someya, Hideki Shigematsu, Tomomi Kimura-Someya, Nipawan Nuemket and Takashi Kumasaka

Table S1 Data-collection conditions for 26 data sets

A total of 26 data sets were collected at SPring-8 BL41XU by using 11 crystals. The data sets for high-resolution data collection are indicated in bold. The 19 data sets collected from 11 crystals were finally merged and are shown on a grey background.

#	Crystal	Data	Beam size (μm)	Wavelength (μm)	Total rotation range (°)	Rotation per image (°)	Exposure time (s/frame)	Thickness of Al filter (μm)	Detector distance (mm)
1	1	1	30	1.0	160	0.5	1	500	400
2		2	30	1.0	30	0.5	1	500	400
3		3	30	1.0	10	0.5	1	500	400
4	2	1	30	1.0	90	0.25	1	700	400
5	3	1	30	1.0	90	0.25	1	600	400
6	4	1	30	1.0	115	0.25	1	600	350
**7**	5	**1**	**50**	**1.0**	**25**	**0.25**	**1**	**200**	**330**
**8**	6	**1**	**50**	**1.0**	**25**	**0.25**	**1**	**300**	**330**
**9**		**2**	**50**	**1.0**	**25**	**0.25**	**1**	**300**	**330**
10		3	50	1.0	25	0.5	1	600	330
11		4	50	1.0	10	0.5	1	700	330
**12**	7	**1**	**50**	**1.0**	**10**	**0.25**	**1**	**200**	**330**
13		2	50	1.0	10	0.5	1	600	330
**14**	8	**1**	**50**	**1.0**	**15**	**0.25**	**1**	**200**	**330**
**15**		**2**	**50**	**1.0**	**15**	**0.25**	**1**	**200**	**330**
16		3	50	1.0	15	0.5	1	650	330
17		1	50	1.0	15	0.5	1	600	330
**18**	9	**1**	**50**	**1.0**	**20**	**0.2**	**1**	**0**	**300**
**19**		**2**	**50**	**1.0**	**10**	**0.1**	**1**	**0**	**300**
20		3	50	1.0	30	0.5	1	400	300
**21**	10	**1**	**50**	**1.0**	**10**	**0.1**	**1**	**0**	**300**
**22**		**2**	**50**	**1.0**	**10**	**0.1**	**1.5**	**0**	**300**
**23**		**3**	**50**	**1.0**	**10**	**0.1**	**2**	**0**	**300**
24		4	50	1.0	30	0.5	1	500	300
**25**	11	**1**	**50**	**1.0**	**10**	**0.1**	**1**	**300**	**300**
26		2	50	1.0	10	0.5	1	300	300
